# Temperature changes in 2% chlorhexidine gluconate using two activation methods with different intensity levels

**DOI:** 10.4317/jced.54732

**Published:** 2018-05-01

**Authors:** Carlos González, Leopoldo Forner, Carmen Llena, Adrián Lozano

**Affiliations:** 1 DDS, Department of Stomatology Universitat de València; 2 MD, DDS, PhD, Department of Stomatology Universitat de València

## Abstract

**Background:**

Chlorhexidine is an irrigant solution widely used in root canal treatment because of its good antimicrobial properties. However, its mixture with sodium hypochlorite results in the appearance of substance called p-chloroaniline, a cytotoxic substance. This is also found when chlorhexidine is heated. The use of passive ultrasonic irrigation is related to an irrigant thermal increase, which can cause the appearance of p-chloroaniline. Thus, the objective is to establish the influence of ultrasonic and sonic activation, with the use of different intensities, upon the temperature of chlorhexidine gluconate (CHX).

**Material and Methods:**

The following groups were established: control (without activation), ultrasonic activation and sonic activation. A 0.5-ml volume of CHX was placed in an Eppendorf tube in a thermo-static bath at 37ºC. Both methods resulted in immediate CHX activation. The temperature was recorded every 30 seconds between 60 and 180 seconds. The statistical analysis was performed based on the Mann-Whitney U-test.

**Results:**

Both groups subjected to activation showed higher temperatures than the control group, and increased activation intensity was associated to greater temperature increments in both activated groups.

**Conclusions:**

Both ultrasonic and sonic activation are correlated to temperature increase in CHX.

** Key words:**Chlorhexidine, p-choroaniline, PUI, sonic irrigation, temperature.

## Introduction

Pulp disease is usually caused by polymicrobial infection, and treatment in such cases involves disinfection and filling of the canal system (1). Sodium hypochlorite is the most widely used irrigant, though this product alone proves insufficient. The addition of a chelating agent and antiseptic such as chlorhexidine is advisable in such situations (2,3). Chlorhexidine (CHX) is commonly used at a concentration of 0.12-2%, with very low toxicity (4). When applied at a concentration of 2%, CHX has important antimicrobial activity (5), with no significant differences versus sodium hypochlorite (6), and also offer substantivity (7). An irrigation protocol based on chlorhexidine was found to reduce the bacterial burden by up to 92% (8). On the other hand, it must be taken into account that the mixture of CHX and sodium hypochlorite produces a cytotoxic precipitate (9-11) containing p-chloroaniline, which is considered to be carcinogenic (Hazardous Substances Data Bank -HSDB-: a database of the National Library of Medicines TOXNET System, 2014). Different irrigant activating systems have been developed in order to improve irrigation efficacy. Passive ultrasonic irrigation (PUI) is used to improve cleaning of the root canals (12,13), and CHX shows increased antimicrobial activity when 

PUI or sonic activation is used (14,15). An increase in irrigant temperature has been reported as a consequence of PUI, and this warming effect moreover increases with the application time (16) or the use of larger ultrasonic tips (17). The incubation of CHX with warming and 95% relative humidity also results in the production of p-chloroaniline (18,19), though only when CHX is warmed to 45ºC and not to 37ºC (9). However, while the association between PUI and irrigant temperature elevation has been described in the literature, and as far as we know, no studies have examined such elevation when vibration-based activating systems such as the Endoactivator® (Dentsply, Tulsa, USA) are used. Recently, new sonic wave irrigant activating tips have been introduced, though to our knowledge, no information is available on their temperature elevating effects.

The present study was carried out to measure the temperature increase of 2% CHX when subjected to ultrasonic and sonic activation, and to establish the influence of different activation intensities. The null hypothesis was that irrigant temperature is not affected in any case.

## Material and Methods

Three groups were established: control (C), ultrasonic activation (US) and sonic activation (S).

In the control group we recorded the temperature without activation every 30 seconds, between 60 and 180 seconds, a total of 10 times (n=10). In the remaining two groups the procedure was as follows: 0.5 ml of CHX was placed in an Eppendorf tube in a thermostatic bath at 37ºC. The irrigant was immediately activated as a result. We recorded the temperature in both groups every 30 seconds, between 60 and 180 seconds, a total of 10 times (n=10), using a TES 1302 type K thermometer (TES Electrical Electron-ic Corp., Taipei, Taiwan). In the US group we used the VDW Ultra ultrasound system (VDW, Munich, Germany) with a 25 caliber IRRI Safe file (VDW, Munich, Germany) at intensity settings of 10%, 20% and 30%. In the S group we used a 25/0.6 polyamide EDDY tip (VDW, Munich, Germany) fitted to a Son-icflex 2003L handpiece (KaVo, Biberach, Germany) with intensity settings of 1, 2 and 3. In this case the thermal changes were recorded after the first 60 seconds.

The mean temperatures and standard deviations were recorded, and comparisons between variables were carried out using the Mann-Whitney U-test, with a 95% confidence interval.

## Results

**Figure 1:**
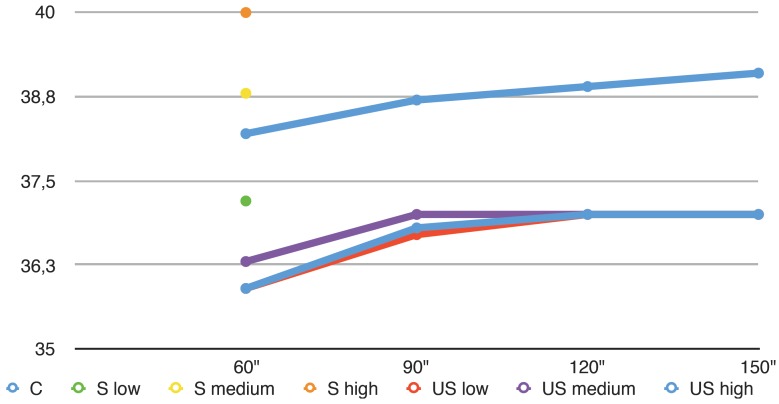
Influence of activation time upon the temperature of the irrigant.

A total of 230 temperature measurements were obtained. The means and standard deviations after 60 and 180 seconds are reported in  Table 1. The time-temperature relationship is shown in Figure 1.

**Table 1:**
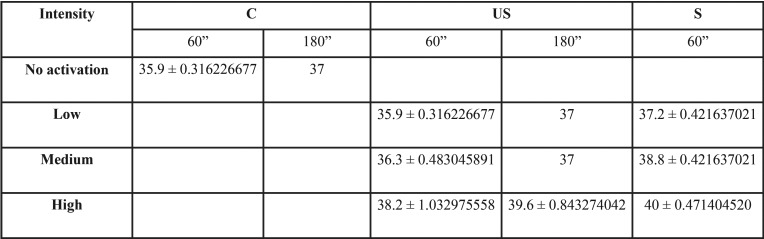
Mean temperature and standard deviation per group after 60 and 180 seconds.

Passive ultrasonic irrigation showed statistically significant differences versus the control group after 60 seconds when the medium and high intensity settings were used (*p*=0.045 and *p*=0.000, respectively). After 180 seconds, statistically significant differences were only observed with the highest intensity (*p*=0.000).

On comparing sonic activation versus the control group after 60 seconds, statistically significant differences were recorded regardless of the intensity setting involved (*p*=0.000). However, comparison after 180 seconds only revealed significant differences at the medium and high intensity settings (*p*=0.000).

Comparisons were made of those variables found to be significantly different from the control values. After 60 seconds, ultrasonic activation resulted in a lesser temperature increase at the medium intensity setting than sonic activation, independently of the intensity used (*p*<0.05). High intensity ultrasonic activation resulted in a greater temperature increase than with the EDDY tip at its lowest intensity setting (*p*=0.012). The increase in turn was similar to that obtained with the EDDY tip at medium intensity (*p*=0.128), but less than that obtained with the sonic activation tip at maximum intensity (*p*=0.000).

## Discussion

A number of studies have associated irrigant temperature elevation to the use of PUI (16,17). On the other hand, such temperature elevation has been related to the appearance of p-chloroaniline as a CHX degradation subproduct (9,18,19). Temperature rise after PUI application could suppose the appearance of these molecules. Furthermore, PUI favors CHX penetration within the dentinal tubules (20), which thereby act as a potential p-chloroaniline reservoir. In contrast, sonic activation has not been found to increase irrigant temperature – though such evidence comes from use of the Endoactivator® (Dentsply, Tulsa, USA). In this regard, the new sonic irrigation tips operate at 6000 Hz, with features similar to those of PUI, such as cavitation or acoustic transmission, and to our knowledge no data have been published to date on their possible effects upon irrigant temperature.

In the present study we found that activation both with PUI using IRRI Safe files and with sonic activa-tion using EDDY tips resulted in CHX temperature elevation. Passive ultrasonic irrigation showed significant differences versus the control group at the medium and high intensity settings, though in the former case only after 60 seconds. In turn, higher intensities were associated to greater temperature increments. However, greater temperature elevations with longer application times were only observed at the maximum intensity setting, since the temperatures recorded after 180 seconds with the medium and low intensities were comparable to those obtained in the control group. In the case of sonic activation, the temperature elevations were significantly greater than in the control group at all intensity settings after 60 and 180 seconds, except for the low intensity setting, where the temperature difference only proved significant after 60 seconds.

An increase in intensity setting does not raise the instrument frequency (set at 6000 Hz) but increases the vibration amplitude of the tip. This suggests a relationship between vibration amplitude and temperature elevation. On comparing the two activation techniques we found the greatest temperature increase to correspond to sonic activation at the highest intensity setting. However, after 60 seconds PUI at high intensity induced a significantly greater temperature increase than the EDDY tip at low intensity, and a temperature increase similar to that obtained with the EDDY tip medium intensity. This suggests that the lowest intensity settings should be chosen with both techniques in order to reduce the impact of activa-tion upon temperature.

A review of the literature has yielded no publications allowing comparisons of the results obtained with sonic activation using EDDY tips. However, previous studies with PUI have reported temperature incre-ments higher than those obtained in our study (16,17). These differences may be attributable to meth-odological discrepancies, however. An *in vivo* study has shown that irrigant solutions balance their temperature with that of the surrounding environment, and a similar phenomenon may occur in our study with the surrounding thermostatic bath (21).

Attending to the temperature, p-chloroaniline has been detected in 0.2% and 2% CHX incubated at 36.5ºC (18,19). However, another study in which 2% CHX was warmed, recorded p-chloroaniline at 45ºC but not at 37ºC (9). Given the results obtained in our work, this substance could appear after activation with the systems studied.

Conduction of the experimental part of our study using Eppendorf tubes may be regarded as a limitation in view of the volume of liquid used (greater than the root canal volume), though this is an easily reproducible way of obtaining homogeneous and comparable results. The temperatures obtained could condition the appearance of p-chloroaniline, as seen in earlier studies (18,19). However, the sonic activation time recommended by the manufacturer is 30 seconds, which may result in a lesser temperature increment. Nevertheless, recording with the new sonic activation tips was carried out after one minute of activation in order to compare the results versus those obtained with PUI. We likewise have found no studies on the efficacy of the EDDY tip with different activation times or intensity settings.

The results obtained in our study show that both ultrasonic and sonic activation produce temperature increments with respect to the control group. The greatest temperature increase was recorded with the highest intensity setting in both activation groups. Sonic activation resulted in similar or greater temperature increments than in the ultrasonic activation group, depending on the intensity setting used. Based on the results obtained, it would be advisable to reduce both the activation time and the intensity. Further studies on the efficacy of these instrument tips and on the relationship between activation and CHX temperature elevation and the appearance of p-chloroaniline are needed.
